# Study on the Influence of Defects on Fracture Mechanical Behavior of Cu/SAC305/Cu Solder Joint

**DOI:** 10.3390/ma15144756

**Published:** 2022-07-07

**Authors:** Sinan Zhang, Zhen Wang, Jie Wang, Guihua Duan, Haixia Li

**Affiliations:** 1 School of Physical Science and Technology, Lanzhou University, Lanzhou 730000, China; zhangsn2018@lzu.edu.cn; 2 State Key Laboratory for Nonlinear Mechanics (LNM), Institute of Mechanics, Chinese Academy of Sciences, Beijing 100190, China; ghduan@lnm.imech.ac.cn; 3 Department of Engineering Mechanics, Tsinghua University, Beijing 100084, China; wangjie19@mails.tsinghua.edu.cn; 4 Institute of Process Engineering, Chinese Academy of Sciences, Beijing 100190, China; hxli@ipe.ac.cn

**Keywords:** Cu/SAC305/Cu solder joints, in situ tensile test, fracture analysis, X-ray μ-CT, FE simulation

## Abstract

The fracture behavior of the Cu/Sn-3.0Ag-0.5Sn (SAC305)/Cu solder joint was investigated by conducting tensile tests with in situ X-ray micro-computed tomography (μ-CT) observation, and finite element (FE) simulation. The tensile fracture process of solder joints with a real internal defect structure was simulated and compared with the experimental results in terms of defect distribution and fracture path. Additionally, the stress distribution around the defects during the tensile process was calculated. The experimental results reveal that the pores near the intermetallic compound (IMC) layers and the flaky cracks inside the solder significantly affected the crack path. The aggregation degree of the spherical pores and the angle between the crack surface and the loading direction controlled the initiation position and propagation path of the cracks. The fracture morphology indicates that the fracture of the IMC layer was brittle, while the solder fracture exhibited ductile tearing. There are significant differences in the fracture morphology under tensile and shear loading.

## 1. Introduction

The failure of a solder joint is a problem that must be urgently addressed in the manufacturing industry of electronic and microelectronic products. Defects such as pores and microcracks in solder joints are a main cause for the low reliability of modern electronic equipment [1,2]. The defects reduce the conductivity and thermal conductivity of solder joints and they reduce the mechanical properties of solder joints owing to the stress concentration [3,4]. Numerous studies have shown that the formation of defects in solder joints is caused by outgassing, metallization defects, and poor solder wettability during the reflow process of the solder and flux [5]. Currently, environmentally benign Pb-free solders (such as SAC305) are widely used [6,7]. However, compared with the Sn–Pb solder, the wettability and spread ability of the Pb-free solder are poor; therefore, the pores and microcracks in the solder joints are more severe [8,9]. Although many technologies can be taken to minimize them, such defects are still inevitable [6,10,11]. Therefore, it is very important to investigate the influence of Pb-free solder defects on the mechanical properties of solder joints, which will help in improving the overall reliability of electronic products.

The SAC305 alloy is the most important solder material, and its mechanical properties have attracted widespread attention. To date, studies have conducted several types of tests, including uniaxial tension tests [12], creep tests [13], and fatigue tests [14], and investigated the influence of solder materials on the mechanical properties from such viewpoints as temperature, strain rate, and load type. However, compared with solder alloys, solder joints often have more complex internal defect structures and welding interfaces, which result in their mechanical properties being different to those of solder alloys. Most studies have reported that the mechanical properties and reliability of solder joints do not only depend on the size of the internal defects, but also on the defect location and distribution [15,16]. The reason for this is that the three-dimensional (3D) morphology and microstructure of defects are different, and the stress concentration distribution is also different. Hence, different mechanical responses are provided under external loading. Currently, the position, size, and distribution of the internal defects in solder joints cannot be predicted in advance [17,18]. Establishing a relationship between the initial internal defect structure of solder joints and their mechanical properties is very important for predicting the service life and improving the reliability of electronics.

Existing studies have conducted extensive numerical evaluations, but have mainly predicted the mechanical behavior of solder joints using a finite element (FE) model without internal defects [19,20] or by randomly setting the size and distribution of internal defects [21,22]. Obviously, there is a certain gap between the simulation results and the actual situation, and it is impossible to compare the FE results with the actual defect evolution process so as to confirm the reliability of the FE results. The X-ray micro-computed tomography (μ-CT) method is a 3D, non-destructive, and quantitative method, and is used to characterize the internal structure of alloys. For opaque materials, this method is very advantageous with regard to the inspection of defects such as inclusions, cracks, and pores, and its resolution has greatly improved with the development of micro-focus X-ray μ-CT technology. Therefore, more detailed information about the microstructure, such as the position, geometry, and distribution of pores, can be obtained quantitatively and with good accuracy [23]. Studies have also attempted to evaluate the mechanical properties of structures with actual defects. Liu et al. [24] characterized the initial internal defect structure of the die-cast AlSiMgMn alloy by X-ray μ-CT, and established an FE model of the actual structure. Their results indicated that the FE simulation can predict the fracture process of the AlSiMgMn alloy, and the results have good consistency. As it is important to understand the 3D microstructure of solder joints, the FE simulation can be used to investigate the fracture behavior of solder joints so as to accurately predict the failure behavior and service life of the solder joints [25,26].

Currently, many related studies have been carried out in the fields of electronics and microelectronics. For example, X-ray μ-CT has been used to quantitatively characterize the number and volumes of pores [27,28], the crack length [29,30], and their distribution in solder joints [31]. This non-destructive method enables us to investigate the damage evolution of internal defects in solder joints under external loading, so as to elucidate the role of the microstructural characteristics in the damage process. Chawla et al. [32] characterized the damage evolution of pores in SAC307 solder joints during a shear experiment by CT interruption (ex situ), and the failure of solder joints was satisfactorily predicted by combining the CT and FE simulation. Gan et al. [33] characterized the propagation of a fatigue crack in a SAC305 solder ball under rapid thermal shock by conducting a CT interruption experiment. Although CT-based ex situ loading experiments have made great progress in characterizing the defects of solder joints, the unloading effect is inevitable during ex situ characterization, such as crack closure or the change process of pore size and volume caused by shrinkage during cooling. Therefore, it is difficult to accurately track the evolution process of microstructural features under external loading, and the relationship between them, only by ex situ CT characterization [34]. In situ experiments based on X-ray μ-CT are gradually developing. Lall et al. [35] investigated the influence of pore morphology on the mechanical properties of solder joints by conducting a three-point bending experiment using in situ X-ray μ-CT. Subsequently, they also measured the thermo-mechanical deformation of solder joints in flexible and rigid printed circuit assemblies [36]. Shi et al. [37] used X-ray μ-CT for the in situ characterization of the microstructural evolution of the reflow cavity in an SAC305 solder joint under shear ratchet fatigue. Although these studies have great significance for the investigation of the mechanical properties of solder joints, they are still insufficient for understanding the failure mechanism and improving the reliability of solder joints. Therefore, it is very urgent to investigate the mechanical properties and internal structure evolution of solder joints by conducting in situ X-ray μ-CT experiments.

In view of the present research situation, this paper would set up an in situ tensile system under μ-CT to study the 3D fracture behavior of solder joints. Based on the self-built tensile platform, a uniaxial tensile experiment of Cu/SAC305/Cu solder joints was conducted at room temperature. The internal defect evolution process of the solder joint was observed during the tensile process, and was quantitatively analyzed by considering the volume changes of different defects and other parameters. The crack propagation path and failure mode were analyzed using high-resolution scanning electron microscopy (SEM) and CT technology. Finally, based on the FE model with initial internal defects, their evolution and the path of crack propagation were predicted. The experimental and calculated results were compared, and the effect of defects on the stress distribution and crack growth is discussed in this paper. Obviously, this fracture prediction method of solder joints with an initial defect structure would provide technical support for predicting the reliability of microelectronic products.

## 2. Experimental Procedure

### 2.1. Sample Preparation

**Figure 1:**
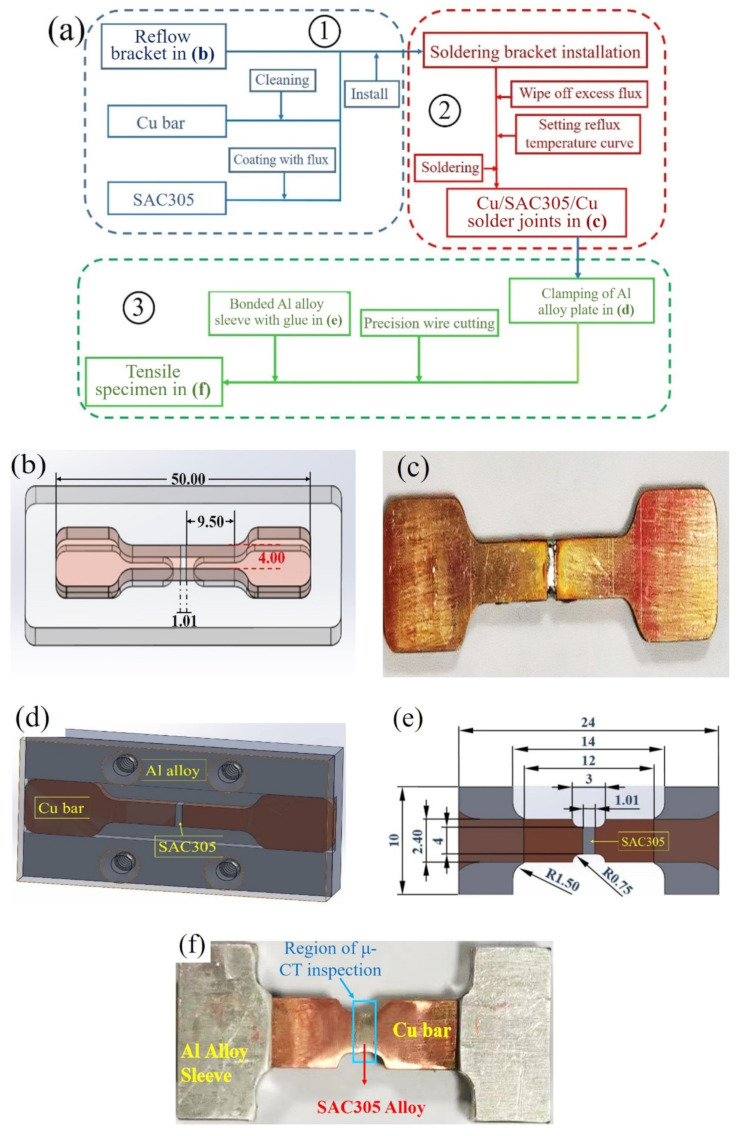
(**a**) The flow diagram of tensile specimen preparation (①: Materials preparation for soldering;②: soldering process;③: tensile specimen processing); (**b**) reflow bracket; (**c**) Cu/SAC305/Cu solder joint; (**d**) self-designed clamp of Al alloy; (**e**) specimen size and geometry in this experiment (unit: mm); (**f**) in situ uniaxial tensile specimen.

The solder used in the experiment was the commercial SAC305 alloy, its nominal composition was displayed in Table 1. To investigate the tensile fracture properties of Cu/SAC305/Cu solder joints, the flow diagram of tensile specimen preparation was shown in Figure 1a, which mainly included three steps. Firstly, two 99.9% Cu bars with a surface roughness, Ra = 1.6 μm, and SAC305 alloy were installed in the specific reflow soldering bracket, which was designed as shown in Figure 1b. The thickness of the substrate was 2.00 mm, the width of its middle position was 4.00 mm and the length was 50.00 mm. The surface of the Cu bar was cleaned before soldering. The cleaning method was to put the Cu bars into solution for cleaning PCB (QL-C7006, ZHEJIANG QLG, Wenzhou, China), and perform ultrasonic oscillation for 8 s. The SAC305 solders and the two bars were coated with flux on both sides, and the excess flux was wiped off. Secondly, according to the requirements of the industrial lead-free soldering reflow temperature curve, the soldering bracket was put into a commercial reflow oven (HERLLER 1809-MK3, HELLER INDUSTRIES, Florham Park, NJ, USA) with a soldering temperature of 265 °C [38]. After soldering, the solder joint was polished using SiC sandpaper with grit scales of 800 and 3000, respectively. The harvested specimens are shown in Figure 1c. The transverse area of the solder joints was 4.00 × 2.02 mm2, and the thickness was approximately 1.01 mm.

Finally, to satisfy the size requirements of the tensile setup and X-ray image, the uniaxial tensile specimen was designed in a dog-bone shape. During the manufacturing process, the solder alloy and the Cu bars were fixed by the self-designed clamp of the Al alloy, as shown in Figure 1d. Precision wire cutting was used, and the geometric dimensions of the tensile specimen were in Figure 1e. To prevent the specimen from pulling off from the grips of the tensile machine, the clamping end of the specimen was wrapped by an Al alloy sleeve, and the clamping end and Al alloy sleeve were bonded with the glue, as shown in Figure 1e. Finally, the uniaxial tensile specimen was obtained, as shown in Figure 1f.

### 2.2. Pore Inspection with X-ray μ-CT

**Figure 2:**
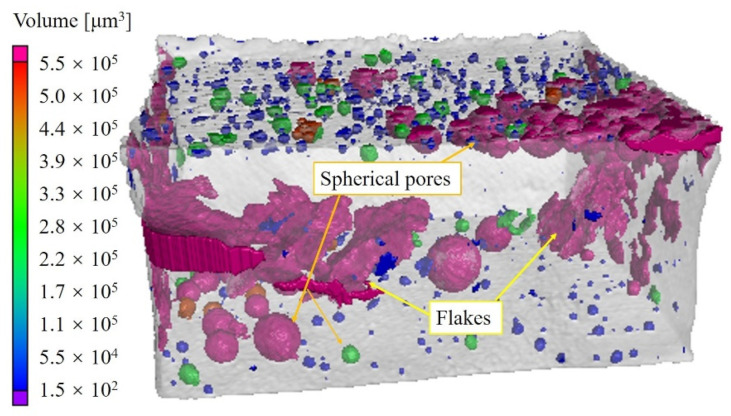
Reconstructed structure of defects in the Cu/SAC305/Cu solder joints.

A 3D X-ray μ-CT system (XRadia, 520versa, ZEISS, Oberkochen, Germany) with a resolution of 1.00 μm was used to obtain the internal microstructure of the solder joints. The inspection area was indicated by the blue rectangle in Figure 1e. The scanning parameters of the X-ray μ-CT were optimized by considering the quality of the scanning images and the unloading effect caused by suspending the experiment. During the test, the voltage and current were 150 kV and 67 μA, respectively. The size of the X-ray raw tomography images was 2048 × 2048 pixels. To obtain a clear image of the internal structure of the pores, the specimen was scanned at 180°, and 501 images were obtained in total. Therefore, the resolution of obtained image was 5.46 μm/pixel. The exposure time of each projection image was approximately 10 s, and the process lasted for approximately 2.5 h. The defect distribution of the Cu/SAC305/Cu solder joint was reconstructed using the VG studio software, as shown in the Figure 2. The different colors of the defects in the Figure 2 indicated the volume of each defect. The geometric shapes of defects were mainly divided into spherical shape and flat shape, which were called spherical pores and flakes, respectively. The spherical pores were mainly distributed in the upper and lower layers of solder joint, namely IMC layers; flakes were mainly distributed inside the solder. The formation mechanism of defects with two different shapes would be explained in detail in Section 4.1.

### 2.3. Experimental Setup and Method

**Figure 3:**
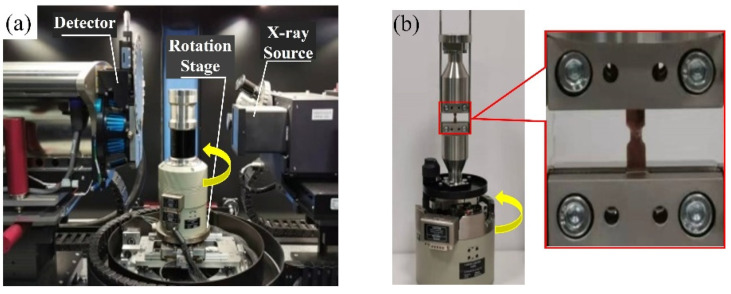
(**a**) Setup of in situ X-ray μ-CT; (**b**) tensile setup capable of rotating with μ-CT test piece table.

To observe the evolution of the internal defects during the tensile process, a CT5000 Deben (Judges Scientific PLC, London, UK) tensile setup was placed in the X-ray μ-CT scanning environment cavity, which was installed on the CT rotatable test table, as shown in Figure 3a. Therefore, the experimental test pieces could rotate by 360°, as shown in Figure 3b, which satisfies the scanning requirements of sample structure reconstruction by the μ-CT. The experiment was carried out in an atmospheric environment. In this experiment, the displacement loading mode was selected, and the loading rate was 0.03 mm/min. When the test piece was in the pre-load state and the displacement was 0.0279 mm and 0.1359 mm, the experiment was suspended and the X-ray μ-CT process began to work, respectively. The load and displacement of the test piece were recorded by the in situ testing machine.

### 2.4. FE Simulation

**Figure 4:**
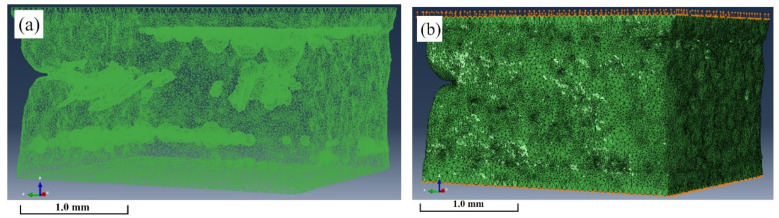
(**a**) Three-dimensional FE mesh model considering real internal defects; (**b**) boundary conditions and displacement loading of FE model.

A FE model with real internal defects was established to simulate the tensile fracture process of solder joints. First, the CT scanning data of the solder joint were imported into the Avizo software to generate the internal structure model. Considering that larger defects and defects near the boundary greatly influence the fracture of the material, in the FE modeling process, the defects with a volume larger than $3.00\times 10^{5}$ μm^3^ were retained. The obtained FE mesh model is shown in Figure 4a. The model was divided into volumetric tetrahedral meshes, and 449,168 elements were generated in total. Finally, the meshes were imported into the FE software for model pre-processing, model calculation, and post-processing analysis. In the process of uniaxial tensile testing, the lower surface of the model was completely fixed, the upper surface was set to displacement loading, and the displacement in the Z direction was set to 0.20 mm, as shown in Figure 4b. This study focused on the influence of internal defects on the fracture behavior of solder joints; therefore, the Cu bars, IMC layers, and microstructure were not considered in the modeling process.

The material property of the SAC305 alloy was set to isotropic hardening. The material parameters were mainly obtained from the experimental tensile curve (see the tensile curve in the Section 3.2, and the fracture strain was set to 2.77%. As the SAC305 alloy has excellent toughness, the ductile damage model was selected. Displacement damage evolution was adopted, and the displacement at failure was $u_{f}^{p}=L\varepsilon_{f}^{p}$, where *L* was the length of the element and $\varepsilon_{f}^{p}$ was the equivalent plastic strain at failure. Since the length of the element was quite wide, for simplicity, the average value was used as the characteristic length of the element, L = 12.67 μm, in our simulation. The value of $\varepsilon_{f}^{p}$ = 12.15% was obtained from the tensile curve. The detailed calculation method could be found in Abaqus Analysis User’s Guide [39]. The degradation of the material stiffness was typically expressed by the scalar damage variable, D, and $\dot{D}={\dot{u}}^{p}/u_{f}^{p}$. When $u^{p}=u_{f}^{p}$, this indicated the failure of the element (D = 1). Subsequently, this element was deleted, which means that the crack was propagating forward.

## 3. Results

### 3.1. Initial Microstructure

**Figure 5:**
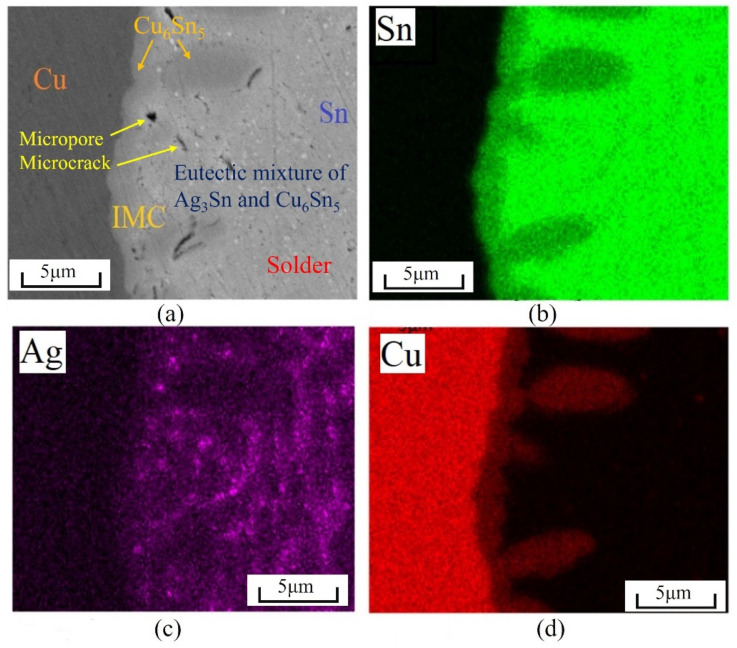
Microstructure and EDS mapping of surface of SAC305/Cu interfaces after polishing. (**a**) initial microstructure; (**b**) Sn element; (**c**) Ag element; (**d**) Cu element.

The main matrix of solder SAC305 was the β-Sn phase, and a large amount of the eutectic mixture of Ag3Sn and Cu6Sn5 was dispersed in it [40]. After the reflow soldering process, the Cu bars at both ends had a metallurgical reaction with solder SAC305. The Cu element in the Cu bars diffused to one side of the solder, and thus formed an IMC layer [2]. Figure 5 shows the microstructure of the solder joint and the EDS mapping of the different elements. The EDS analysis revealed that the scallop-IMC layer was formed along the interface, which was mainly in the Cu6Sn5 phase. As the solder joint was not subjected to age treatment in this experiment, the Cu3Sn phase did not form a thick-layered structure in the SAC305/Cu interface, similar to the Cu6Sn5 phase [11]. Additionally, various micropores and microcracks appeared in the IMC layer and its vicinity; their influence on the welding strength is explained in detail in Section 4.1. On the right side of Figure 5a, many Ag-rich white particles can be observed in the solder matrix. The white particles represent the Ag3Sn phase, which indicates brittleness [1].

### 3.2. Uniaxial Tensile Test Results

**Figure 6:**
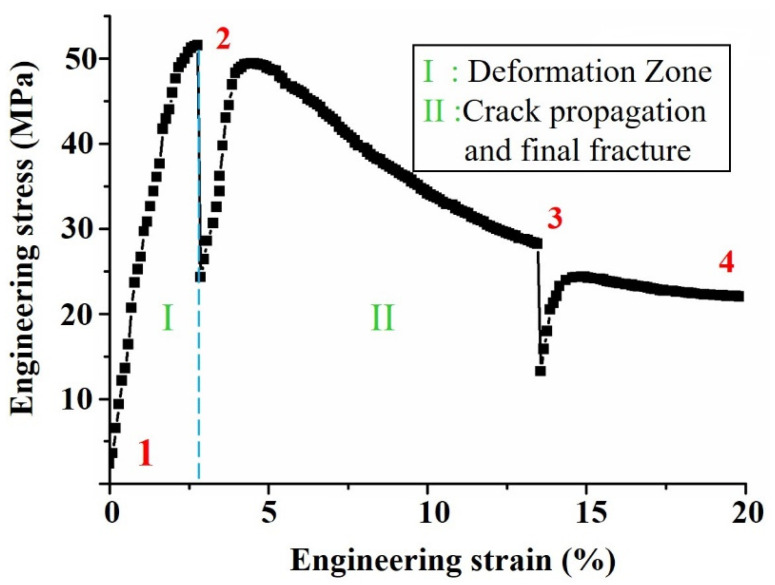
Engineering stress–strain curve of Cu/SAC305/Cu solder joint obtained by in situ tensile test. Positions 1, 2 and 3 represented the suspending points, and 4 represented the fracture point.

Figure 6 showed the engineering stress–strain curve of the Cu/SAC305/Cu solder joint, which was obtained from the load–displacement data of the testing machine. As could be seen from the curve, the fracture strength of the solder joint was 51.52 MPa, which was very close to the tensile strength of 55.00 MPa of the SAC305 alloy [41]. In this experiment, the gap size of the two Cu bars was 1.01 mm. Zimrich et al. [42] reported that, as the solder gap increased, the fracture strength of the solder joint gradually approached the tensile strength of the solder. Additionally, as could be seen from the curve in Figure 6, the tensile process of the solder joints could be divided into the deformation zone (I) and the crack propagation and final fracture zone (II). For both zones, the internal deformation of the material was discussed in Section 3.3. Moreover, the fracture strain of the solder joint reached 19.87%, which was mainly related to the good toughness of the SAC305 alloy [43].

### 3.3. Fracture Process and Fracture Morphology

**Figure 7:**
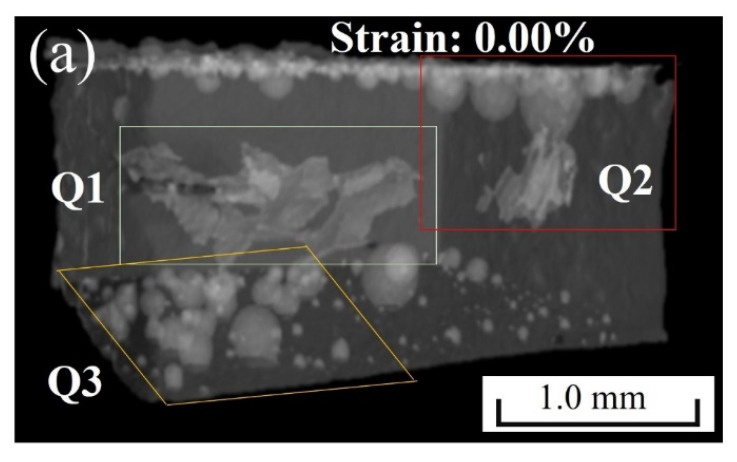

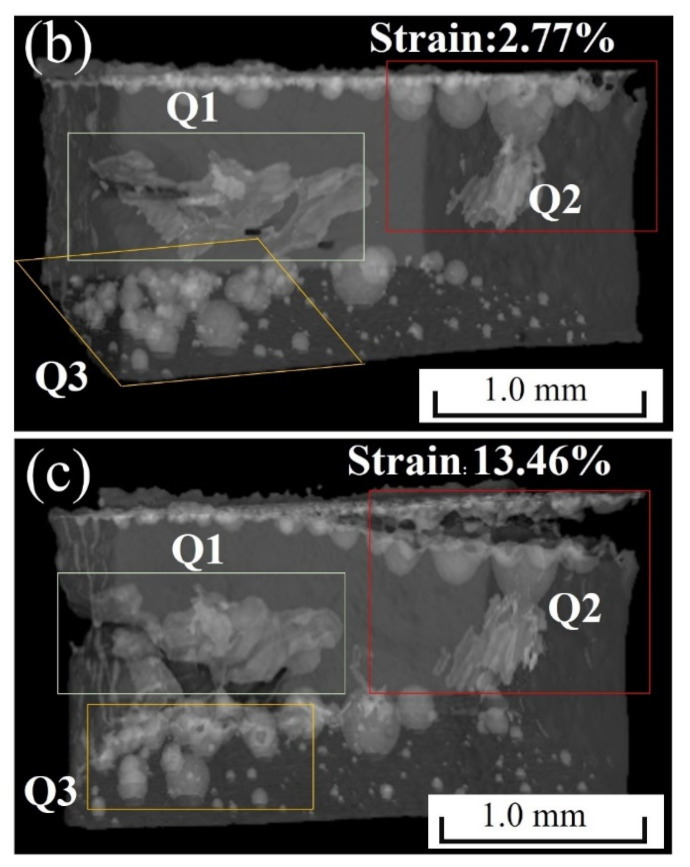
Three-dimensional internal defects in microstructure of solder joints at the strain of: (**a**) 0.00; (**b**) 2.77%; (**c**) 13.46%.

Figure 7 showed the 3D defect evolution of the solder joints during the tensile process at room temperature. The upper and lower boundary layers were the IMC layers connected to the Cu bars. The microstructure of the internal defects at the initial state was shown in Figure 7a. As could be seen, a flake defect existed in the middle Q1 region. Moreover, many pores were produced in the Q2 and Q3 regions near the IMC layer. The formation mechanism of the two types of defects was explained in detail in Section 4.1. The spherical pores in the Q2 region were connected at the upper IMC. When the deformation reached 2.77%, the flakes at the Q1 region opened and propagated forward. A spherical defect in the upper right corner of the Q2 region was torn. However, there was no obvious change in the separated pores in the Q3 region. When the strain was 13.46%, the defects in the Q1 region penetrated longitudinally and connected with various spherical pores in the Q3 region. The defects in the Q2 region formed cracks, which propagated forward along the IMC layer to the middle of the solder joint. Under continuous tension, the solder joint finally broke.

**Figure 8:**
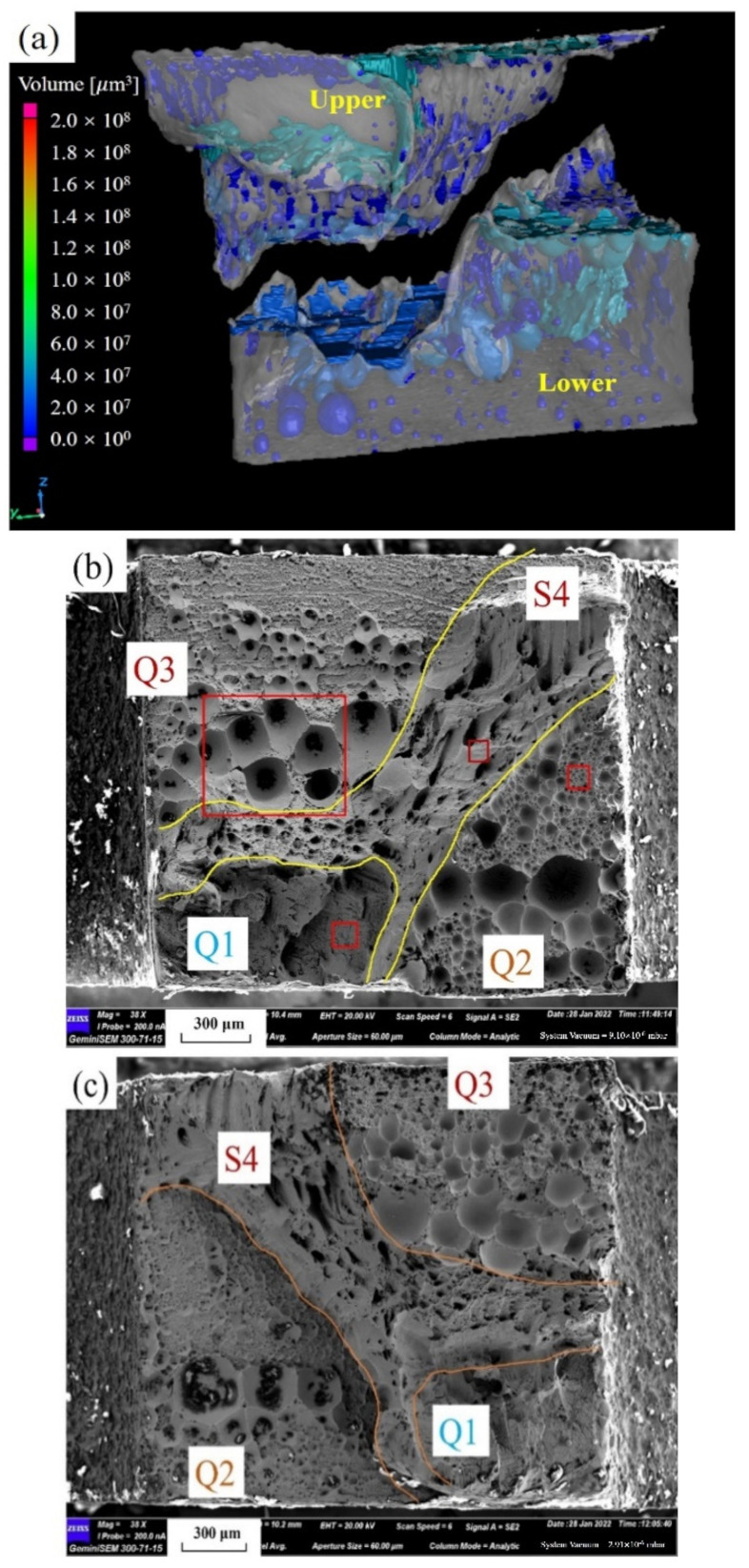
(**a**) Reconstruction of 3D internal structure of solder joint after fracture; (**b**) SEM fracture morphology of lower part; (**c**) SEM fracture morphology of upper part.

Figure 8a showed the 3D CT reconstruction morphology of the solder joints after the fractures. As could be seen, the final fracture morphology of the solder joint was relatively complex, and included the fracture surface of two IMC layers and the solder material itself. Figure 8b,c showed the fracture morphology of the lower and upper parts, respectively. According to the characteristics of the fracture morphology, it can be determined that a tensile fracture occurred in the Q1, Q2, and Q3 regions in Figure 7, and the fracture surface was perpendicular to the loading axis. They corresponded to the Q1, Q2, and Q3 regions in Figure 8b,c, respectively. However, since the fracture surfaces of Q1–Q3 in Figure 7 were not on the same plane, the S4 fracture surface was formed due to the shear action. Under the action of the shear force, the defects in this region underwent severe deformation. Obviously, there were significant differences between the tensile fracture and shear fracture.

### 3.4. Evolution Process of Defects by In situ *X-ray* μ-*CT*

**Figure 9:**
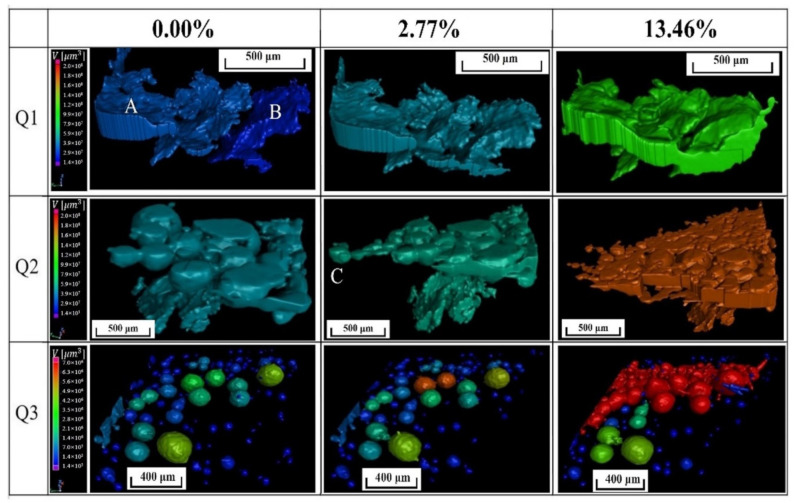
Evolution of 3D defects as strain increased in three regions of solder joints.

To quantitatively describe the evolution of the internal pores during the tensile process of the Cu/SAC305/Cu solder joints, the defects in three regions (Q1, Q2, and Q3), respectively, were extracted as shown in Figure 9. In the Q1 region, two flake defects mainly existed, and were marked as A and B, respectively. Defect A was connected to the outer surface, and defect B was an internal defect. At the initial state, the two defects in region Q1 were separated. When the deformation reached 2.77%, defects A and B connected. When the deformation was 13.46%, the thickness and volume of the defect significantly increased. Region Q2 was an aggregated pore defect close to the IMC layer. The pores partially connected in the soldering process. As the strain increased to 2.77%, the pores continued to spread forward and “swallowed” a small pore (C). When the strain reached 13.46%, the crack propagated further, and the propagation direction was perpendicular to the loading. Region Q3 consisted of aggregated spherical pores near the IMC layer. However, it could be seen that these pores were not connected and remained in a separated state. When the strain reached 2.77%, the spherical pores grew, but an obvious connection phenomenon did not exist. When the strain increased to 13.46%, some spherical pores began to connect and aggregate.

**Figure 10:**
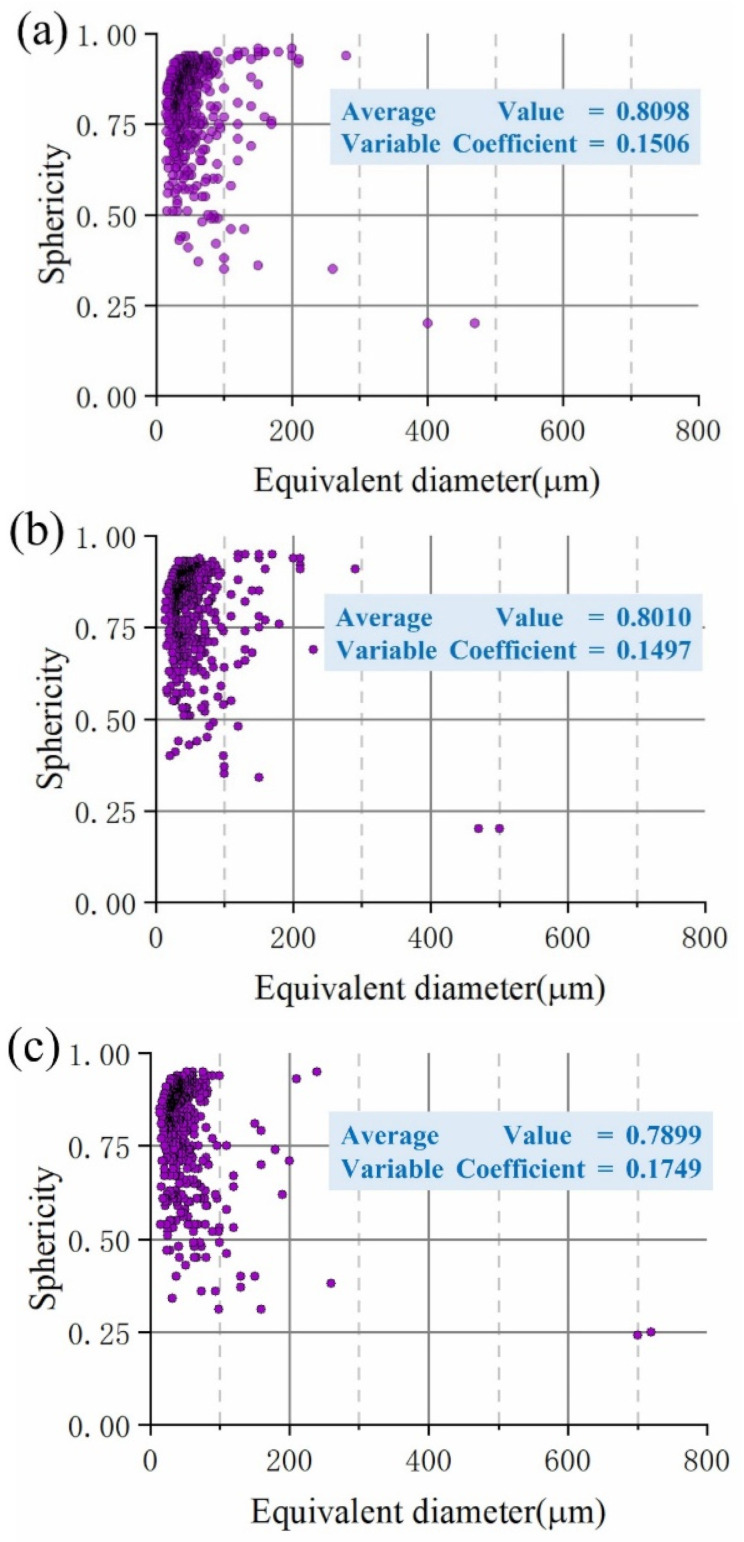
Sphericity distribution of defects at solder joints under different deformation states: (a) 0.00; (b) 2.77%; (c) 13.46%.

The volume and shape evolution of the internal defects were quantitatively characterized. Generally, the shape characteristics of the defects were characterized by the sphericity parameters, that is, the ratio between the spherical surface, which was the same as the volume of the defect, and the defective surface [32]. Figure 10 showed the sphericities of the defects in the solder joint under different strain conditions. As could be seen, the average sphericity decreased as the deformation increased, which indicated that the defect shape was changing. The variation of the normalized dispersions (variable coefficient) in the crack propagation and final fracture zone (II) was greater than that in the deformation zone (I). The total volume, general surface, and volume ratio of the defects were also quantitatively characterized, as presented in Table 2. As can be seen, as the deformation gradually increased, the growth rate of the total volume, the general surface area, and the defect volume ratio of the defects in the crack propagation were greater than those in the deformation zone.

### 3.5. Simulation Results

**Figure 11:**
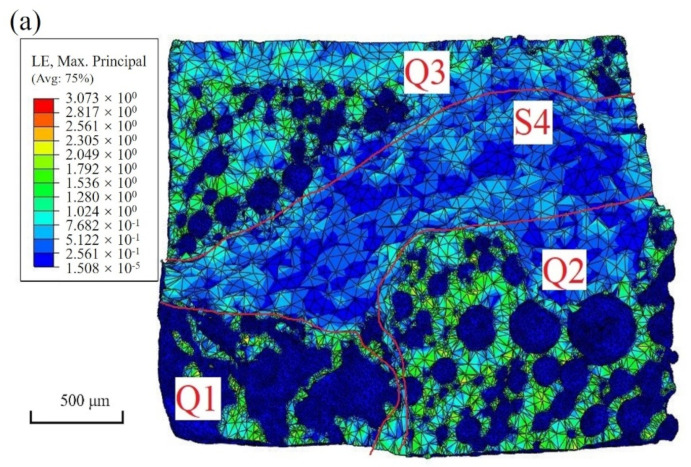

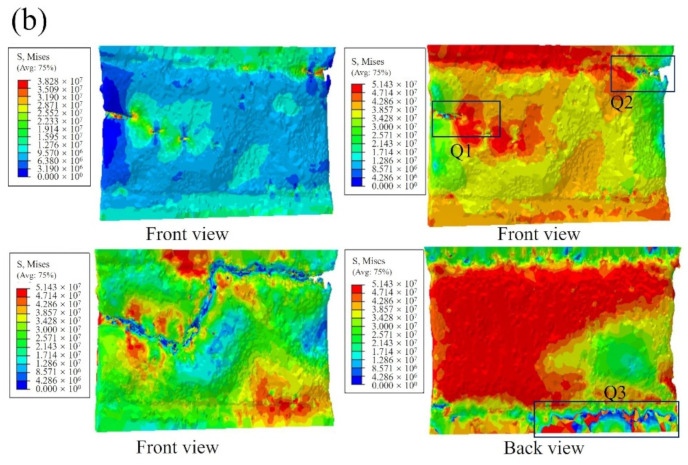
FE simulation results: (**a**) fracture morphology of solder joints; (**b**) process of crack initiation and propagation for solder joints.

Figure 11 showed the simulation results for the fracture surface and crack growth. As shown in Figure 11a, the fracture morphology was divided into four zones: Q1, Q2, and Q3 were tensile fracture zones, and S4 was a shear fracture zone, similar to the experimental results shown in Figure 8b. Figure 11b showed the simulation results for the crack propagation process under different displacement loading conditions, which revealed that the crack was first initiated and propagated from the defects in regions Q1 and Q2. However, the crack in region Q2 was propagated faster along the direction perpendicular to the loading direction during the continuous tensile process. The reliability of the simulation results was verified as shown in Figure 8c. In summary, the propagation path and the rate of cracks were closely related to the distribution of the internal defects of the solder joints, as discussed in detail in Section 4.1.

## 4. Discussion

### 4.1. Defects Affecting Crack Initiation and Propagation

Defects were inevitable for solder joints [11]. As shown in Figure 2, there were many spherical pores in the upper and lower boundaries (the IMC layer and its vicinity) of the reconstructed solder joints, and most of these pores were mutually separated. Generally, it was considered that the main causes of the internal defects of solder joints are as follows: (1) the additive or organic impurities remaining in the Cu bar; (2) the poor wettability of the solder; and (3) the exhaust of the solder and flux during reflow [18,44]. In this study, after the surface of the Cu bars was cleaned by solution for cleaning PCB, a small amount of water and organic material remained on its surface. In addition, the flux was a volatile material [10]. Therefore, during the reflow soldering process, a high temperature caused the flux, remaining water, and organic material to evaporate, and then left the molten solder alloy. However, some gases were not discharged, which resulted in some gases remaining in the solder joints to form spherical pores. Many pores were concentrated near the IMCs. Moreover, there were a few pores that did not completely leave the solder. The shrinkage pores and flakes might form near the solder surface after the solder solidifies, similar to the crack in the Q1 region in Figure 7a [10].

**Figure 12:**
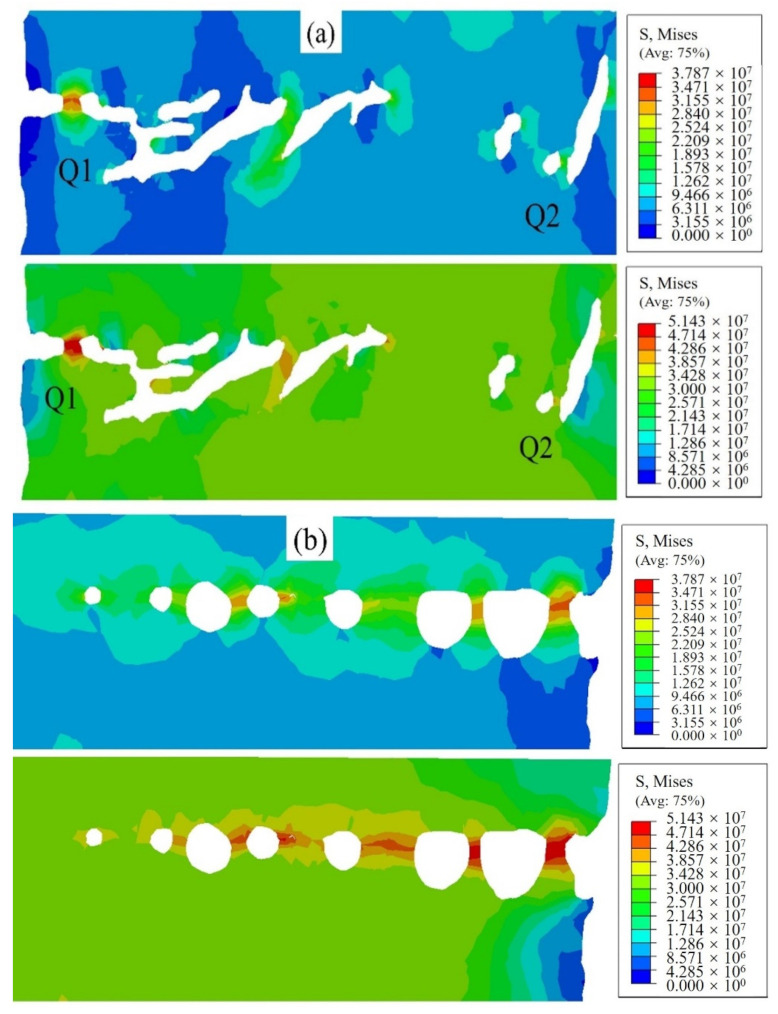
Stress distribution (**a**) near flaky cracks in regions Q1 and Q2 and (**b**) near aggregation pores in region Q2, under different external loads.

The position and shape of defects in the solder joints had a significant influence on the stress distribution. As shown in Figure 2, the pore distribution of the Cu/SAC305/Cu solder joint was very complex. However, only a few very small spherical pores and two flakes existed inside the solder. The flake in region Q1 was perpendicular to the loading axis, while the flake in region Q2 was inside the solder and approximately parallel to the loading direction. Figure 12a showed the stress distribution around the two flake cracks. Obviously, the stress concentration in region Q1 was greater than that in region Q2. Moreover, when the crack tip stress of the flake crack in region Q1 reached the fracture strength, the crack tip in region Q2 did not; therefore, the cracks did not begin to propagate. However, the aggregated pores in region Q2 around the IMC layer cracked during loading. During the subsequent propagation process, the propagation rate was significantly higher than that of the flaky crack in region Q1. To explain this phenomenon, the stress distribution in region Q2 was shown in Figure 12b. Hence, it could be inferred that the effective bearing area was greatly reduced owing to the distribution of the aggregated pores along the IMC layer. Therefore, the aggregated pores in region Q2 connected as the strain increased, which led to the cracking along the IMC layer in region Q2. Additionally, aggregated pores also existed in the lower IMC layer, as shown in Figure 2. By comparing the characteristics of the pores in regions Q2 and Q3, it was found that the average spacing of pores in region Q3 was larger. Therefore, the crack did not initiate from region Q3 of the lower IMC layer. However, in the tensile process, the separated pores constantly grew and connected inside. When the two propagating cracks in regions Q1 and Q2 connected, the aggregated pores in region Q3 also broke. As the two Q2 and Q3 fracture zones were not in the same cross-section, the S4 shear fracture region was formed. Therefore, it was understood that the fracture path of the solder joints was controlled by the shape and distribution of defects.

### 4.2. Fracture Mechanism of Solder Joints

**Figure 13:**
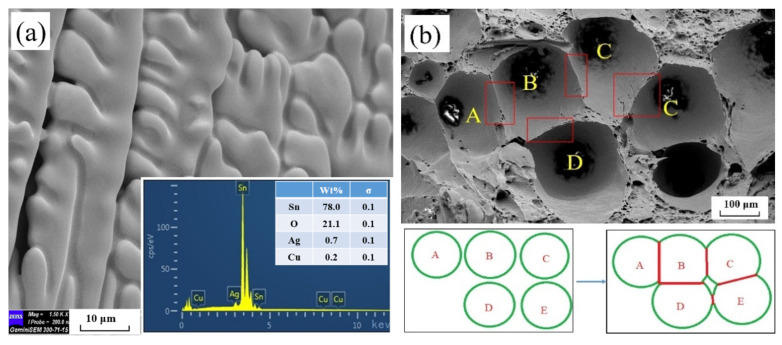

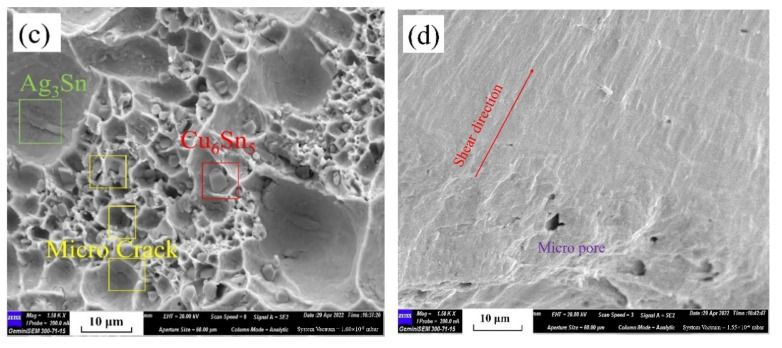
Partially enlarged image: (**a**) Q1 region; (**b**) pores in Q3 region; (**c**) fracture surface of IMC layer in Q2 region; (**d**) S4 region.

As shown in Figure 8 of Section 3.3, the fracture morphology exhibited different characteristics in different regions. To better explain the fracture mechanism of the solder joint, the characteristic positions of different regions in Figure 8b were partially enlarged. Figure 13a showed an enlarged view of the flake surface in the Q1 region, and it could be seen that the smooth surface implies recrystallization characteristics. The formation mechanism of flakes in the Q1 region was effectively demonstrated. In regions Q2 and Q3, there were some spherical pores with diameters ranging from tens of microns to more than one hundred microns, and the partially enlarged view was shown in Figure 13b. As could be seen, owing to the small space among the pores, the final fracture morphology had pores with partial edges that were characterized by tearing, as shown in the red box in Figure 13b. Additionally, it was found that the tearing morphology of a single pore was polygonal, and its specific shape characteristics were related to the distribution of the surrounding pores. Under tensile stress, the pore spaces were continuously necked, owing to a large plastic deformation. When the stress exceeded the fracture strength, the solder alloy fractured and formed a linear tear pattern, as indicated by the red line in Figure 13b. However, the edges of pores without adjacent pores retained an arc-shaped tear pattern, as indicated by the green arc line in Figure 13b. Additionally, the crack propagation path in the IMC layer was perpendicular to the direction of the loading axis, and was a Mode I crack, as shown in Figure 7c. When the strain reached 13.46%, its propagation path length was longer than that of the crack in region Q1. The main reason for this was that the plastic deformation of the SAC305 alloy consumed a lot of the external work, owing to the great toughness [12]. However, the IMC layer was brittle, which meant that the external work in the IMC layers was almost converted into fracture energy. Therefore, the fracture area and propagation rate of the IMC layers were much larger compared to those in region Q1.

**Figure 14:**
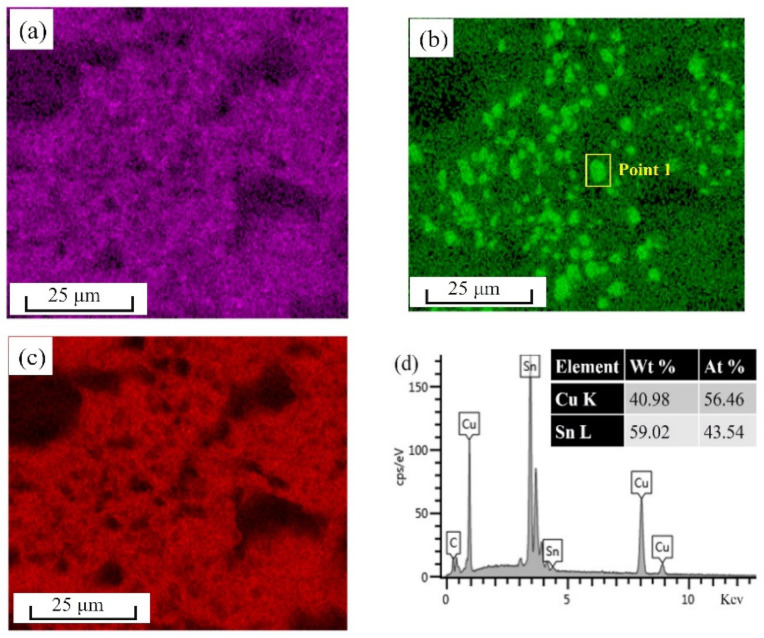
EDS mapping of fracture surface of IMC layer in Q2 region: (**a**) Ag element; (**b**) Cu element; (**c**) Sn element; (**d**) component ratio of prism particles at point 1.

Additionally, in the partially enlarged image in Figure 8b, it can be seen that there were many microstructural characteristics besides the pore defect evolution in Figure 13c. First, the component distribution in Figure 13c was characterized by EDS mapping. The distributions of Sn, Cu, and Ag elements in the fracture surface were shown in Figure 14a–c, respectively. In Figure 14a, some small and bright dots were relatively evenly distributed in the whole area. However, the Cu element distribution in Figure 14b was not uniform, and the positions of the bright spots were consistent with those of the prismatic particles in Figure 13c. In order to determine the material type of the particles, quantitative analysis was performed on the particles at point 1 in Figure 14b, as shown in Figure 14d. Thus, from the component ratio, it could be determined that the prism particles are the Cu_5_Sn_6_ phase, which proved that the fracture surfaces of regions Q2 and Q3 were IMC layers. Overall, the IMC layers were brittle, and had many pores around them, which was the main reason why the IMC layers became the weak link between the SAC305 solder and the Cu bar. Another reason was that a large concentration of stress existed on the lower and upper IMC layers, owing to the aggregation of the pores. Additionally, interface debonding between the Cu_6_Sn_5_ particles and the β-Sn matrix was observed on the fracture surface, as shown in Figure 13c. Tang et al. [45] reported that the elastic modulus of the Cu_6_Sn_5_ phase was approximately three times equal to that of the β-Sn matrix. Therefore, the deformation between the Cu_6_Sn_5_ phase and the β-Sn matrix was inconsistent during the tensile process. Additionally, various microcracks were formed on the fracture surface owing to the large local stress caused by the inconsistent deformation. As mentioned earlier, the fracture morphology of the S4 region was mainly the shear area, and the partial enlarged view is shown in Figure 13d. Since it mainly existed inside the SAC305 alloy, there were no prismatic particles. The shearing direction could be determined from the streamline in Figure 13d.

## 5. Conclusions

This study used in situ X-ray μ-CT to quantitatively and visually characterize the evolution of the internal defects of Cu/SAC305/Cu solder joints under uniaxial tensile loading. The influence of the location, distribution, and aggregation of the defects on the fracture behavior of the solder joints was characterized by CT images. The FE model was established using the CT reconstruction data of the solder joints at the initial state. Based on the experimental and simulation results, the main conclusions drawn from this study are as follows:

(1) The tensile strength of the Cu/SAC305/Cu solder joint is 51.52 MPa, which is close to the fracture strength of the solder materials, and its fracture strain is 19.87% due to the great toughness of the solder material.
(2) In terms of the crack propagation path, the simulation results are in good agreement with the experimental results. The reliability of the simulation results obtained by this study was verified by the in situ X-ray μ-CT observation of the 3D crack path.
(3) The fracture morphology of the solder joints can be divided into four regions, namely, regions Q1, Q2, and Q3, which comprise the tensile fracture zone, and region S4, which is the shear fracture zone. As region Q2 is a brittle IMC layer and contains aggregated pores, its crack propagation rate was significantly higher compared with region Q1.
(4) In the tensile fracture zone of Q2 and Q3, on one side of the solder, the tearing ridges of the aggregated pores were linear, owing to the large deformation, while those on the other side were arc-shaped. Additionally, the interface between the Cu_6_Sn_5_ phase and the β-Sn matrix debonded, owing to the inconsistent deformation.

Although some results had been obtained in the fracture research of the Cu/SAC305/Cu solder joint, there were still some shortcomings, which should also be the prospect of future work. In microelectronics, the service of solder joints was mainly caused by the synergy of electrical current and mechanical load, so the influence of electrical current on fracture behaviors could not be ignored. On the other hand, due to the limited resolution of μ-CT, some microstructures, such as the Cu_6_Sn_5_, Ag_3_Sn phase in IMC layers, could not be established in the FE model, thus the accuracy of the simulation results needs to be further improved.

## Figures and Tables

**Table 1:** The nominal composition of SAC305 solder alloy (wt.%).

Elements	Sn	Ag	Cu
SAC305 (wt.%)	96.50	3.00%	0.50

**Table 2:** Evolution of total internal defects under different deformation states.

Strain State	0.00%	2.77%	13.46%
General surface of defects (μm2)	14.31 × $10^{6}$	15.26 × $10^{6}$	20.16 × $10^{6}$
Total volume of defects (μm3)	2.02 × $10^{8}$	2.26 × $10^{8}$	4.60 × $10^{8}$
Defect volume ratio	4.11%	4.59%	8.31%

## Data Availability

All data are presented within the manuscript.
